# Asymptomatic Severe Acute Respiratory Syndrome–associated Coronavirus Infection

**DOI:** 10.3201/eid0911.030401

**Published:** 2003-11

**Authors:** Harold K.K. Lee, Eugene Y.K. Tso, T. N. Chau, Owen T.Y. Tsang, K, W. Choi, Thomas S.T. Lai

**Affiliations:** * Princess Margaret Hospital, Hong Kong

**Keywords:** Severe Acute Respiratory Syndrome, SARS, coronavirus, SARS-CoV, Hong Kong, asymptomatic, seroconversion

**To the Editor**: An outbreak of severe acute respiratory syndrome (SARS) began in Hong Kong in March 2003. As of May 29, 2003, a total of 1,732 cases were confirmed; 381 case-patients were healthcare workers and medical students. Clinical features, treatment protocols, and outcomes have been previously reported by various local experts (*1**–**3*). The etiologic agent is a SARS-associated coronavirus (SARS-CoV) (*1*). However, no asymptomatic case of SARS-CoV infection has been previously reported (*4*). In addition, in Hong Kong, blood donors have not shown any detectable antibody to SARS-CoV (*1*). We report a case of possible asymptomatic SARS-CoV infection in Hong Kong.

The case-patient is a registered nurse working in Princess Margaret Hospital, the major infectious diseases hospital that treated >600 SARS patients in Hong Kong. Within this hospital, >800 frontline staff members have participated in direct care of SARS patients, and SARS developed in 62 of these staff members. All healthcare workers working in SARS wards followed the same infection control measures, wearing a N-95 respirator, eye shield, disposable cap, water-resistant gown, and gloves. Gowns and equipment were removed before the staff left the SARS wards.

We performed serologic testing of the first 101 healthcare workers (doctors, nurses, healthcare assistants) who worked in the SARS wards but in whom SARS did not develop. The serologic testing was performed 7–8 weeks after the healthcare workers were first exposed to SARS patients. We identified a nurse who was asymptomatic for SARS-CoV infection, worked in the SARS ward since the disease outbreak, and used full infection control procedures as recommended by the World Health Organization (WHO). The nurse performed procedures, including nasopharyngeal aspiration, handling of fecal matter, and oral feeding of SARS patients. SARS developed in six colleagues who worked in the same ward. She had unprotected exposure to a colleague who contracted SARS and required hospitalization. Serologic testing for SARS-CoV antibody was performed in the microbiology laboratory of Princess Margaret Hospital on week 8 of the nurse’s SARS ward duty. The result of the test was positive by enzyme-linked immunosorbent assay. The test was repeated by the Government Virus Unit of the Department of Health, one of the reference laboratories in Hong Kong. The second test also showed a positive result with an antibody titer of 400 by immunofluorescence assay (normal: <25). We performed another serologic test on week 10 of her SARS ward duty; the result was again positive. The nurse was interviewed by two physicians and questioned about her health condition since February 2003. She did not report any symptoms typical of SARS, such as fever, chills, rigors, malaise, myalgia, cough, dyspnea, and diarrhea (*1**,**3*) during and after her SARS ward duty. She did have a mild, short-term headache, which she has had periodically for many years. She did not take sick leave since February 2003. She did not record any rise in body temperature >37°C and had a leukocyte count of 5.9 x 10^9^/L and a lymphocyte count of 1.6 x 10^9^/L. Results of liver and renal function tests were all normal. Reverse transcription-polymerase chain reaction results for SARS-CoV in stool, urine, throat, and nasal swabs collected during weeks 10 and 14 of her SARS ward duty were all negative. No abnormal radiologic change was identified in the lungs. She lived with four family members and had close contact with them. None of her family members contracted SARS, and all showed a negative result in the serologic testing for SARS-CoV.

We think that asymptomatic and subclinical infection of SARS-CoV exists and can result in seroconversion; however, this kind of asymptomatic seroconversion is probably uncommon. Why a person infected with SARS-CoV did not have typical symptoms, and the infectivity of an asymptomatic person is unknown. A person’s genetic makeup may determine susceptibility to SARS-CoV and the final clinical outcome. We agree with Seto et al. (*5*) that recall bias is a concern. However, recall bias probably had little effect since the events took place recently. Moreover, the hospitalization of the nurse’s infected colleague would have made her more alert and aware of symptoms of the illness.
